# CT-based machine learning radiomics modeling to screen for lumbar spine osteoporosis

**DOI:** 10.3389/fmed.2026.1758313

**Published:** 2026-01-23

**Authors:** Cheng Gao, Shu Yang, Jue Zhang, Zhuanghui Wang

**Affiliations:** 1 Department of Orthopedics, The Affiliated Jiangning Hospital with Nanjing Medical University, Nanjing, Jiangsu, China; 2 Department of Cardiology, The Affiliated Taizhou People’s Hospital of Nanjing Medical University, Taizhou, Jiangsu, China; 3 Department of Orthopedics, The First Affiliated Hospital of Nanjing Medical University, Nanjing, Jiangsu, China

**Keywords:** CT, lumbar spine, machine learning, osteoporosis, radiomics

## Abstract

**Objectives:**

Undiagnosed osteoporosis before spinal surgery increases severe complication risks. This study develops the machine learning-based CT radiomics model to preoperatively screen lumbar osteoporosis.

**Materials and methods:**

This retrospective study enrolled 166 patients undergoing concurrent dual-energy X-ray absorptiometry (DEXA), spinal CT and MRI. Vertebral data from normal and osteoporotic cases were partitioned into training and validation cohorts (8:2 ratio). A total of 851 radiomics features were extracted from lumbar spine CT scans using the 3D slicer PyRadiomics module. Feature selection employed mRMR (minimum redundancy maximum relevance) for preliminary screening followed by LASSO regression for dimensionality reduction. Four machine learning classifiers were developed: logistic regression (LR), support vector machines (SVM), XGBoost, and random forest (RF). Model performance was assessed through receiver operating characteristic (ROC) analysis with DeLong test comparisons. Clinical utility was quantified via decision curve analysis (DCA).

**Results:**

Nine radiomic features based on spine CT images were constructed to develop the model. The radiomic-XGBoost model with the highest area under the curve (AUC) of 0.89 of the training cohort and 0.91 of the test cohort among the machine learning algorithms. The DeLong test showed that the differences between the radiomic-XGBoost, vertebral bone quality (VBQ) and Hounsfield unit (HU) models were statistically significant (*p* < 0.05). DCA revealed that the radiomics-based model offers a superior net benefit compared to the other two models.

**Conclusion:**

CT-based machine learning radiomics significantly outperformed VBQ scoring and HU measurements in osteoporosis diagnostic accuracy.

## Introduction

Osteoporosis is a systemic metabolic disorder marked by reduced bone integrity and elevated fracture, predominantly affecting elderly individuals, postmenopausal women, and prolonged hormone therapy recipients (1–3). Global population aging has exacerbated its socioeconomic impact. Given associated severe complications and disability risks, timely diagnosis and intervention are critical (4, 5). However, diagnostic and therapeutic resources remain inaccessible in underserved regions. Dual-energy X-ray absorptiometry (DEXA) is the standard screening tool. Although DEXA itself is low-cost and low-radiation, its routine preoperative use in spinal surgery is limited by the practical hurdle of requiring an additional, dedicated examination (6). Consequently, alternative screening strategies are imperative to identify this critical comorbid condition in elderly populations without DEXA availability.

Previous studies have indicated that computed tomography (CT)-derived Hounsfield unit (HU) values moderately correlate with bone quality and compressive strength (7). Low vertebral HU levels are independently associated with osteoporotic vertebral compression fractures (OVCF) and postoperative secondary fractures in elderly populations (8, 9). However, HU measurements fail to account for cortical bone quality effects on bone mineral density, leading to substantial information loss. A recently developed magnetic resonance imaging (MRI)-based vertebral bone quality (VBQ) score provides enhanced precision in bone quality assessment. Subsequent research validates VBQ’s clinical utility for osteoporosis screening, fragility fracture prediction, and postoperative complication evaluation including cage subsidence, adjacent segment disease (ASD), revision surgery and pedicle screw loosening (10–13). However, the above definitions primarily reflect planar vertebral bone quality without incorporating three-dimensional structural data.

Radiomics, an emerging analytical approach, extracts quantitative imaging features from medical digital images to facilitate clinical decision support through high-dimensional data mining, thereby enhancing diagnostic, prognostic, and predictive precision. Recent research highlights CT- and MRI-based radiomics models demonstrating significant potential for osteoporosis diagnosis (14, 15). Furthermore, integration of radiomics features with machine learning algorithms enables development of disease prediction models (16). Therefore, the present study aimed to extract radiomics features from CT imaging data and investigate the value of radiomics-based machine learning algorithms in detecting osteoporosis.

## Materials and methods

### Study population

This retrospective study received approval from the Institutional Ethics Review Board of our hospital. Consecutive patients were identified by extracting data from the departmental database between January 2020 and June 2023. Initial screening revealed 208 patients who underwent lumbar spine CT, lumbar spine MRI, and DEXA scans within a 30-day interval. Patients were excluded based on the following criteria: (1) trauma, fractures, tumor, infection, (2) ankylosing spondylitis, diffuse idiopathic skeletal hyperostosis, (3) history of prior spinal surgical interventions, (4) inadequate or unclear imaging documentation. A total of 166 patients were included in the final analytical cohort.

Of the 166 patients in this study, 54 were diagnosed with osteoporosis through DEXA. L1–L4 vertebrae from all participants were analyzed, initially enrolling 664 vertebral bodies. After excluding 8 vertebrae due to inadequate region of interest (ROI) identification for analysis caused primarily by imaging artifacts or anatomical overlap, 656 vertebrae were retained, of which 210 (32.0%) exhibited osteoporosis based on DEXA criteria. A stratified random sampling method applied at the patient level allocated the vertebrae into training (*n* = 525, 80%) and test (*n* = 131, 20%) cohorts for radiomics analysis. Clinical variables including age, sex, body mass index (BMI), smoking history, and alcohol consumption were retrospectively extracted from medical records.

### Radiological parameters

All imaging data were obtained through lumbar spine CT and 1.5T MRI. Image analysis utilized integrated digital measurement tools within the Picture Archiving and Communication System (PACS) software. Radiological assessment included the lumbar HU value and MRI-based VBQ score. HU values, validated as strongly correlating with bone mineral density (BMD), were measured using the technique described by Ji et al. (17). Using standard PACS software, an elliptic ROI was drawn on three non-consecutive axial images: inferior to the superior endplate, mid-vertebral body, and superior to the inferior endplate. ROI was defined as a single maximum size ellipse encapsulating only cancellous bone instead of cortical bone. Mean HU values per ROI were calculated by PACS software, with the three-measurement average representing the final vertebral HU value. VBQ scoring was performed on non-contrast sagittal T1-weighted sequences. ROIs were positioned within the medullary compartments of L1–L4 vertebral bodies and the cerebrospinal fluid (CSF) space at L3. In cases of scoliosis or venous plexus obstruction, parasagittal sections were substituted to approximate median sagittal medullary signals. If L3 CSF space was obscured by nerve roots, CSF signals were measured at L2/L4. VBQ scores were calculated as the ratio of median vertebral body signal intensity (L1–L4) to CSF signal intensity (VBQ = SI_vertebrae_/SI_CSF_). Two researchers who were blinded to DEXA *T* values independently assessed HU and VBQ scores, with final values derived from interobserver averages.

### Diagnostic criteria of osteoporosis and image acquisition

All DEXA measurements were acquired using a Hologic Discovery dual-energy X-ray bone densitometer (Hologic Inc., United States) for hip and lumbar spine assessments. The diagnosis of osteoporosis was based on the *T*-score from DEXA scans of the lumbar spine (L1–L4).

Participants were classified into osteoporosis (*T*-score ≤−2.5) and non-osteoporosis (*T*-score >−2.5) groups following World Health Organization (WHO) diagnostic criteria (18). CT and MRI datasets were retrospectively retrieved from institutional PACS, with all images exported in standardized Digital Imaging and Communications in Medicine (DICOM) format.

### Image segmentation and radiomic feature extraction

**Figure 1:**
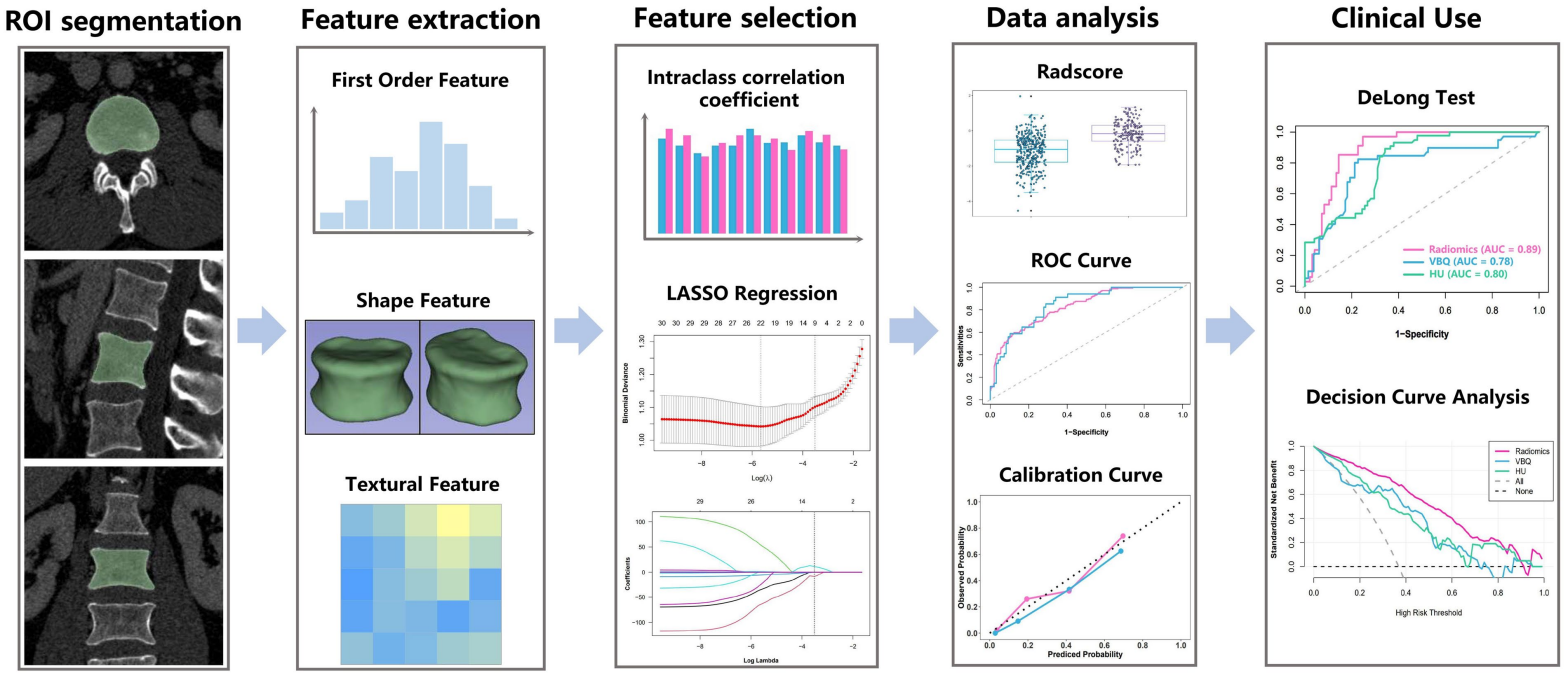
Workflow of the whole research. Image acquisition, processing, radiomic analysis, and modeling pipeline.

The radiomics workflow is schematically presented in Figure 1. All patients underwent lumbar spine scanning using a 64-slice spiral CT, with the scanning parameters as follows: tube voltage 120 kVp, tube current 300 mA, slice thickness 1.25 mm, and slice interval 0.625 mm. Using 3D Slicer (version 5.2.1; www.slicer.org), three-dimensional volumetric segmentations were manually delineated along vertebral body cortical margins and bilateral pedicle anterior boundaries on axial CT datasets. Semi-automated bone segmentation was achieved through automated thresholding and seed propagation techniques. All resulting segmentations were then meticulously reviewed and manually corrected in three planes by two senior musculoskeletal radiologists to ensure anatomical accuracy. Subsequent image standardization and radiomic feature extraction were conducted using Pyradiomics package (version 2.12; https://pyradiomics.readthedocs.io/en/2.1.2/) with the following preprocessing parameters: isotropic resampling (1 × 1 × 1 mm^3^ voxel dimensions) for spatial normalization, followed by intensity discretization with 25 HU bin-width partitioning to reduce image noise and enhance intensity normalization (19).

Following the installation of the PyRadiomics radiomics extension within 3D Slicer, we extracted 851 radiomic features from each three-dimensional ROI, including 162 intensity distribution descriptors (first-order statistics), 14 morphometric parameters (3D shape), and 675 textural biomarkers derived from advanced matrix analyses-specifically 216 co-occurrence (GLCM), 126 dependence (GLDM), 144 run-length (GLRLM), 144 size-zone (GLSZM), and 45 neighborhood gray-tone difference (NGTDM) matrix-based features.

A three-step dimensionality reduction protocol was established to identify robust radiomic features through stability assessment and feature optimization. First, an initial cohort of 50 patients was randomly selected for evaluating feature stability through inter- and intra-observer reliability analyses. To determine inter-observer reproducibility, two radiologists independently performed ROI segmentations in a blinded fashion. To evaluate intra-observer reproducibility, reader 1 repeated the same segmentation process after a one-week interval.

Feature stability was quantified using the intraclass correlation coefficient (ICC), with features demonstrating excellent reliability (ICC >0.90 for both inter- and intra-observer comparisons) being retained for subsequent analysis. Second, the minimum redundancy-maximum relevance (MRMR) algorithm was employed for feature filtering and selection. MRMR ranks features through dual optimization: maximizing mutual information with clinical outcomes while minimizing redundancy among selected higher ranked features (20). The 30 highest-ranking MRMR features were retained for model optimization. Then, the least absolute shrinkage and selection operator logistic (LASSO) regression algorithm, with 10-fold cross-validation was applied to the primary cohort for final feature selection. The penalty parameter (*λ*) was optimized through cross-validation, selecting features with non-zero coefficients. A radiomics signature was formulated as a linear combination of selected features weighted by their respective regression coefficients. Radiomic score (Rad score) for individual vertebral bodies was mathematically derived through the following formula:

$$\sum_{i=0}^{n}C_{i}\times X_{i}+b$$

where *X_i_* is the parameter of the *i*th selected feature, *C_i_* is the coefficient of the *i*th selected feature, and *b* is the intercept.

### Development, validation and clinical utility of predictive models

The radiomics-based predictive models were engineered through systematic implementation of four supervised machine learning paradigms in the training cohort: logistic regression (LR), support vector machines (SVM), extreme gradient boosting (XGBoost), and random forest (RF). The hyperparameters of all machine learning models were optimized to prevent overfitting and maximize generalization performance. We employed a grid search strategy combined with 5-fold cross-validation exclusively on the training cohort. Model performance was quantified via receiver operating characteristic (ROC) analysis, with area under the curve (AUC) comparisons conducted across both training and validation cohorts. Bootstrap resampling (*n* = 1,000) generated calibration curves to evaluate probabilistic concordance between predicted and observed osteoporosis outcomes. The performance of the radiomic signature, VBQ and HU models was assessed using AUC. The clinical utility quantification method employed decision curve analysis (DCA), calculating net benefit differentials across probability thresholds (0–100%) within the independent validation dataset.

### Statistical analysis

Results were expressed as mean ± standard deviation. Continuous data were compared using parametric (Student’s *t*-test) or nonparametric (Mann–Whitney *U*) methods, while categorical variables were evaluated by Pearson’s *χ*^2^ test or exact methods (Fisher’s test), based on data distribution and sample size characteristics. The MRMR algorithm was implemented in R with “mRMRe” package. We used the “glmnet” package to perform the LASSO algorithm. ROC plots and AUC comparison procedures were conducted using the “pROC” package. The “rms” package was used for calibration curves. The DeLong test was then used to compare the ROC curves. DCA was performed using the “rmda” package. Statistical analysis was conducted in R (version 4.2.2, http://www.r-project.org) with significance thresholds set at *p* < 0.05.

## Results

### Patient characteristics

Tables 1, 2 detail the demographic profiles and baseline parameters of the study population. The training and validation cohorts exhibited comparable osteoporosis prevalence rates (33.75% vs. 26.0%; *p* = 0.116). The clinical and radiological characteristics of the train and validation cohorts did not differ significantly. There are differences in age, VBQ score and HU value between the osteoporosis and the normal group in two cohorts.

**Table 1:** Baseline patient characteristics.

Factor	Training cohort (*n* = 525)	Test cohort (*n* = 131)	*p*-value
Age, mean ± SD, years	65.4 ± 12.2	63.6 ± 11.0	0.133
Gender			
Male	225	60	0.554
Female	300	71	
BMI, mean ± SD	24.4 ± 3.1	24.3 ± 3.2	0.650
Smoking status			
Non-smoker	454	106	0.383
Smoker	71	25	
Alcohol intake history			
Yes	152	44	0.291
No	373	87	
VBQ, mean ± SD	3.0 ± 0.8	2.9 ± 0.9	0.904
HU, mean ± SD	114.6 ± 24.6	121.3 ± 25.7	0.075
Radiomics score, median (interquartile range)	−0.753 (−1.585, −0.133)	−0.844 (−1.714, −0.203)	0.254

**Table 2:** Demographic and clinical characteristics in the training and test cohorts.

Characteristics	Training cohort (*n* = 525)			Test cohort (*n* = 131)		
	Osteoporosis	Normal	*p*-value	Osteoporosis	Normal	*p*-value
Age, mean ± SD, years	70.6 ± 10.8	62.7 ± 11.9	<0.001	69.2 ± 10.2	61.7 ± 10.7	0.001
Gender						
Male	54	171	0.002	11	49	0.067
Female	122	178		23	48	
BMI, mean ± SD	24.6 ± 3.4	24.3 ± 2.9	0.470	24.1 ± 3.3	24.4 ± 3.1	0.649
Smoking status						
Non-smoker	151	303	0.675	29	77	0.104
Smoker	25	46		5	20	
Alcohol intake history						
Yes	47	105	0.355	9	35	10.307
No	129	244		25	62	
VBQ, mean ± SD	3.5 ± 0.8	2.7 ± 0.7	0.001	3.8 ± 0.8	2.7 ± 0.6	<0.001
HU, mean ± SD	94.6 ± 25.7	124.7 ± 34.1	<0.001	99.1 ± 22.5	129.1 ± 38.9	<0.001
Radiomics score, median (interquartile range)	−0.136 (−0.537, 0.378)	−1.079 (−1.846, −0.530)	<0.001	0.073 (−0.390, 0.537)	–1.19 (−2.143, −0.471)	<0.001

### Feature selection and radiomics signature construction

**Figure 2:**
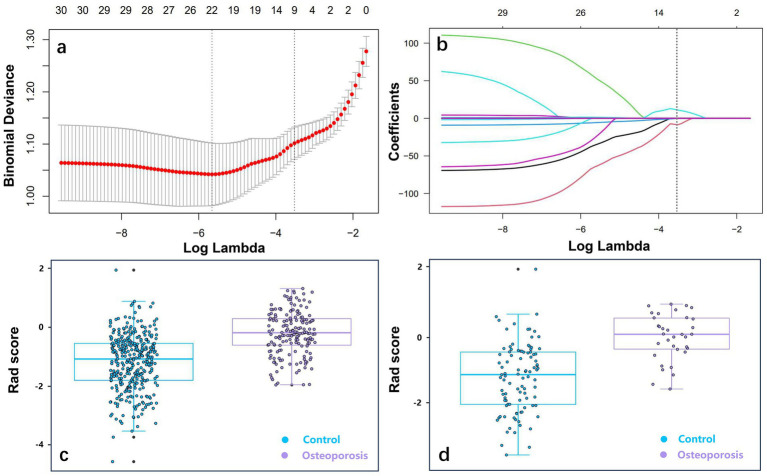
Radiomic feature selection by using LASSO logistic regression. **(a)** Optimal regularization parameter (*λ*) determination through 10-fold cross-validation, with corresponding AUC values versus log (*λ*). Vertical dashed lines indicate optimal *λ* selection based on minimum criteria (*λ*min) and 1 standard error rule (*λ*1se). **(b)** Coefficient trajectories of 30 candidate features, with the vertical line at the optimal *λ* value identifying nine non-zero coefficients for final model construction. Scatter plots of the radiomic score between the control and osteoporosis groups in the training cohort **(c)** and test cohort **(d)**. LASSO, least absolute shrinkage and selection operator; AUC, area under the curve.

Of 851 initial radiomic features, 450 demonstrating high reproducibility (intra-/inter-observer ICCs >0.90) were advanced for subsequent analysis. Next, MRMR algorithm identified 30 non-redundant predictive features, which were subsequently subjected to LASSO regression with tenfold cross-validation (Figure 2). The LASSO algorithm selected nine features with non-zero coefficients to construct the radiomic signature (Table 3). Individual radiomic scores were computed through weighted linear combination of these features. As shown in Table 2 and Figure 2, significant intergroup disparities in radiomic scores were observed between osteoporosis patients (−0.136, 0.073) and non-osteoporosis group (−1.079, −1.19) in the training cohort and test cohort, respectively.

**Table 3:** Radiomic feature final selected by LASSO regression and the coefficient to develop the radiomic signature.

Image type	Feature class	Feature name	Coefficient
Wavelet-LLH	gldm	DependenceNonUniformityNormalized	−0.8532121
Wavelet-HHL	glcm	ClusterShade	0.0123804
Original	firstorder	Skewness	−0.0119066
Wavelet-LHL	glszm	LargeAreaLowGrayLevelEmphasis	−0.0447949
Wavelet-HLH	gldm	DependenceNonUniformity	−0.7171973
Wavelet-LLH	ngtdm	Complexity	0.0275214
Log-sigma-4-0-mm-3D	Glszm	SmallAreaEmphasis	−0.0119066
Wavelet-LLH	Glszm	LargeAreaLowGrayLevelEmphasis	−0.0003290
Wavelet-HHH	glcm	Correlation	1.1146427
Intercept (*β*)	—	—	2.7736518

### Development, performance, and validation of prediction models

**Figure 3:**
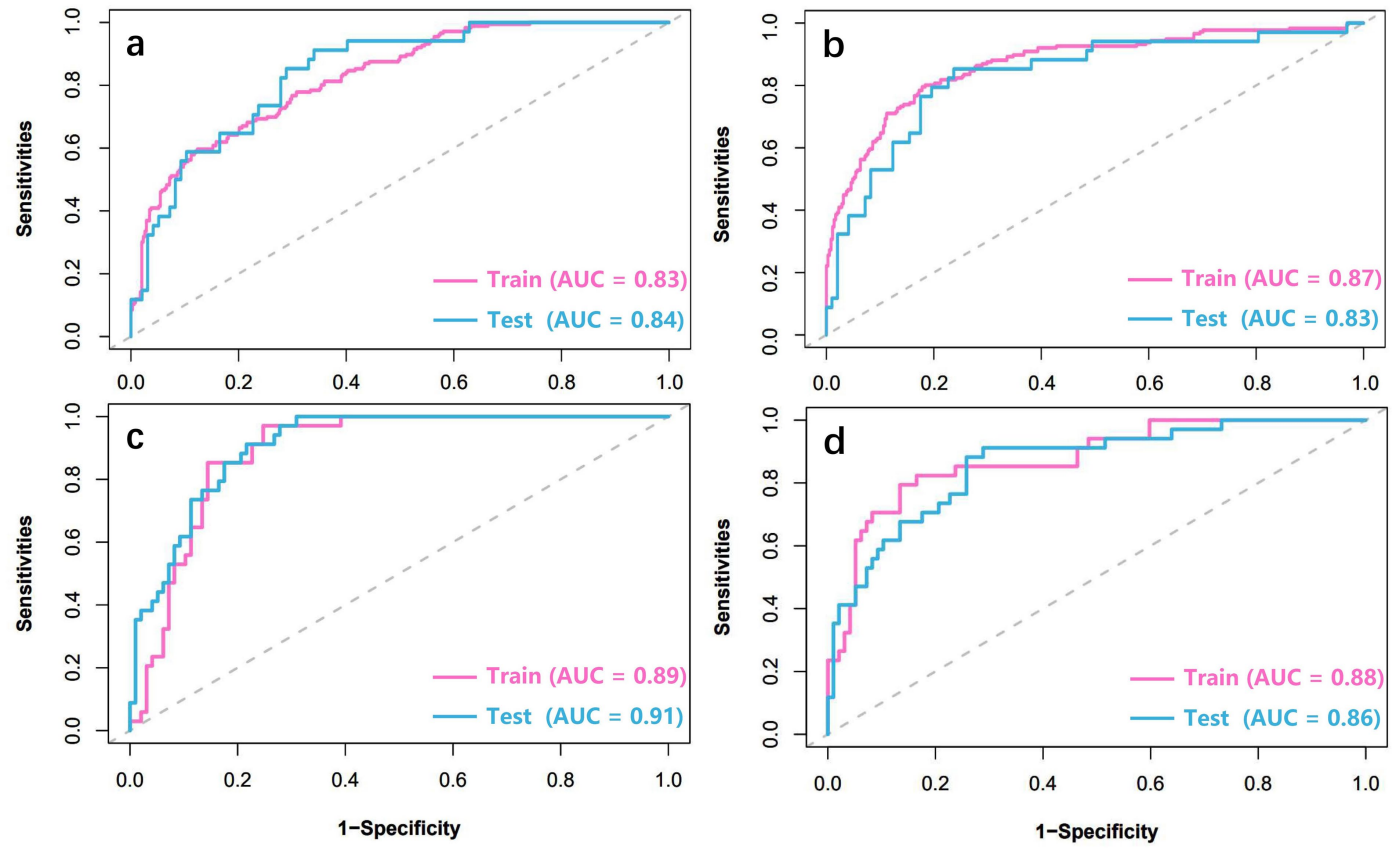
The predictive performance of machine learning models based on the radiomic signature for osteoporosis. The ROC curves are shown for the models of LR **(a)**, SVM **(b)**, XGBoost **(c)**, and RF **(d)**. ROC, receiver operating characteristic; LR, logistic regression; SVM, support vector machine; XGBoost, extreme gradient boosting; RF, random forest.

**Figure 4:**
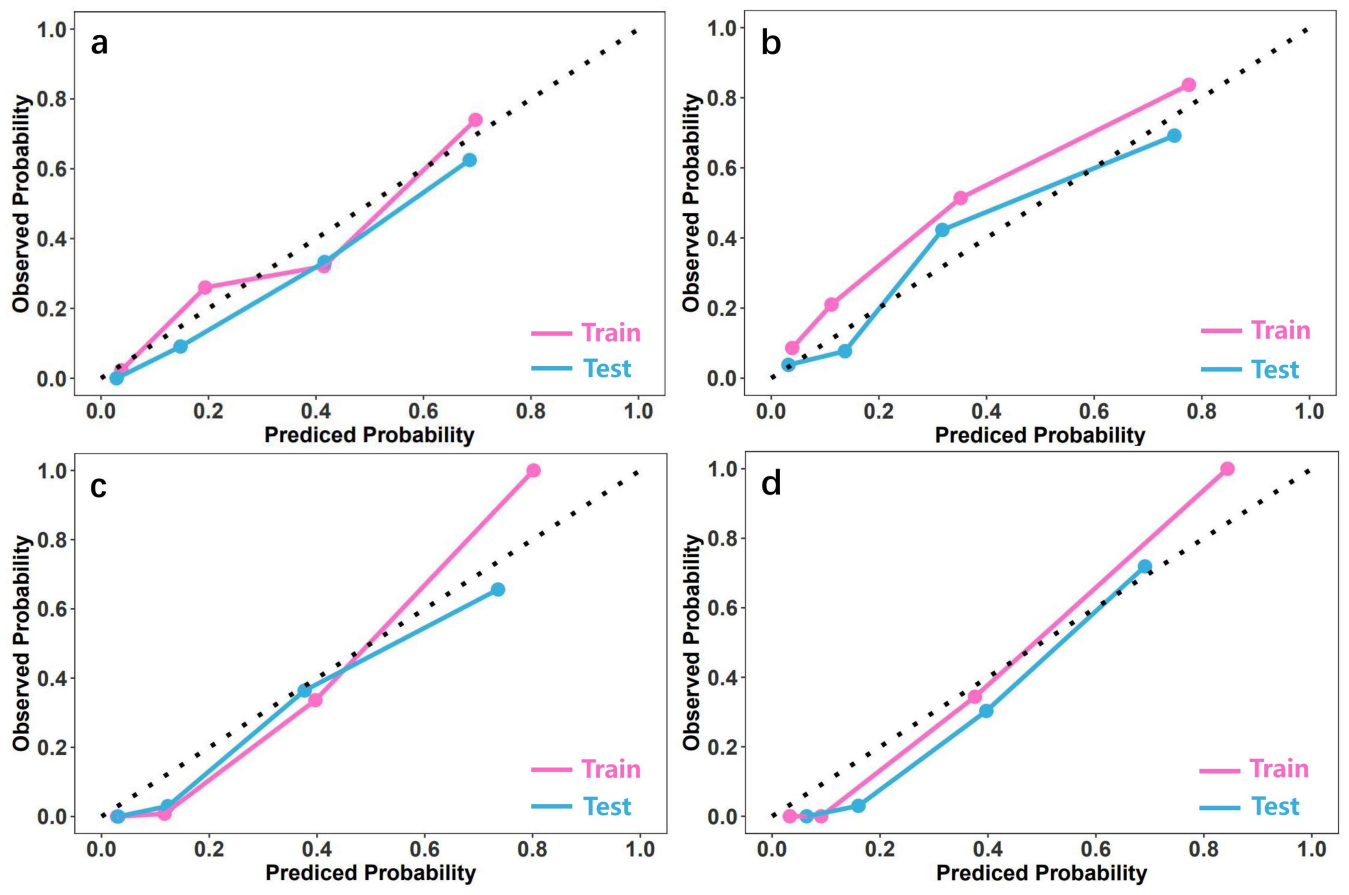
The calibration curves of models for predicting osteoporosis. LR **(a)**, SVM **(b)**, XGBoost **(c)**, and RF **(d)**. LR, logistic regression; SVM, support vector machine; XGBoost, extreme gradient boosting; RF, random forest.

**Figure 5:**
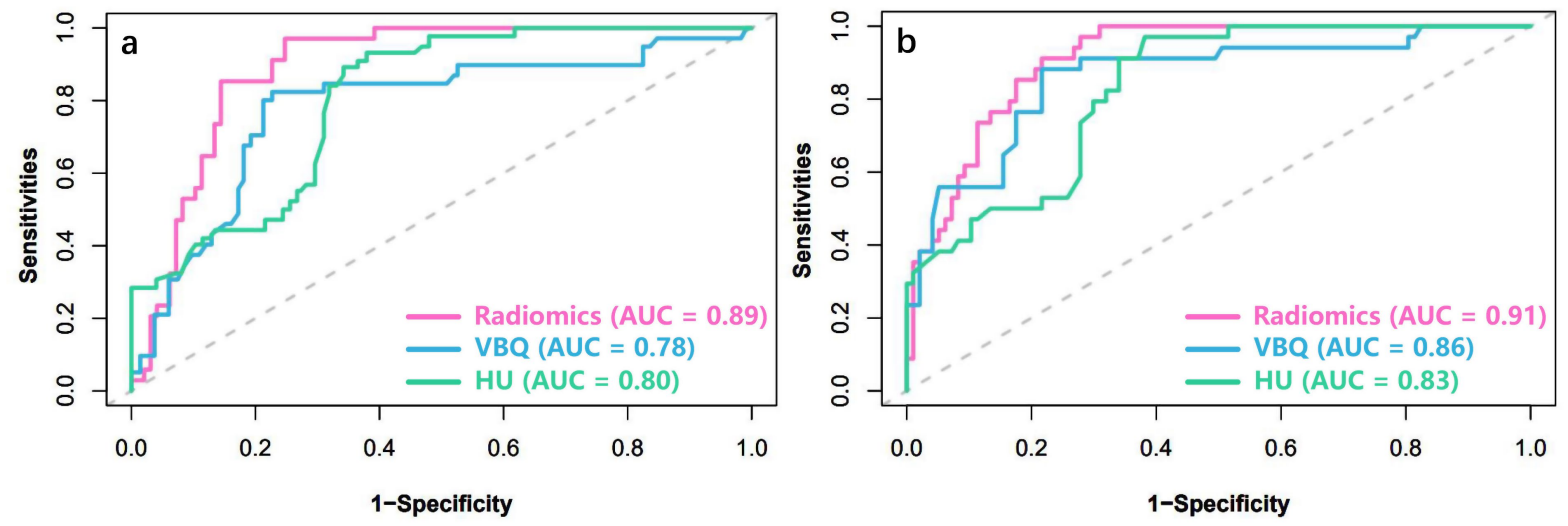
ROC analysis showing that the performance of the radiomics signature model was better than that of the VBQ and HU models in both the training **(a)** and test **(b)** cohorts. ROC, receiver operating characteristic; VBQ, vertebral bone quality; HU, Hounsfield unit.

The ROC curves of four machine learning models (LR, SVM, XGBoost, RF) are shown in Figure 3. For ROC curve of LR, AUC of training set is 0.827, 95% confidence interval (CI) is 0.791–0.863, AUC of test set is 0.842, 95% CI is 0.771–0.913. The AUC of SVM was 0.870 (0.836–0.904) in the training set and 0.834 (0.749–0.919) in the test set. XGBoost shows the best diagnostic performance. AUC of training set is 0.891, 95% CI is 0.837–0.946, AUC of test set is 0.910, 95% CI is 0.863–0.958. RF also has relatively good performance, with an AUC of 0.877 (95% CI: 0.808–0.945) in the training cohort, and an AUC of 0.861 (95% CI: 0.789–0.932) in the test cohort. The calibration curve shows a high degree of fit with the ideal curve (Figure 4). Furthermore, in both the training and testing cohorts, the radiomics-XGBoost model achieved high sensitivity (0.875 and 0.893), specificity (0.859 and 0.824), positive predictive value (0.806 and 0.829), and negative predictive value (0.921 and 0.938), indicating that the radiomics-XGBoost model has good predictive ability for osteoporosis. The radiomic-XGBoost model further compares with the radiographic parameters including VBQ score and HU value on the training and test cohorts to verify its predictive ability. The ROC curves of the radiomics, VBQ and HU models are illustrated in Figure 5. The DeLong test suggested that machine learning radiomics model was more effective than VBQ and HU model in predicting osteoporosis (*p* < 0.05).

### Clinical use

**Figure 6:**
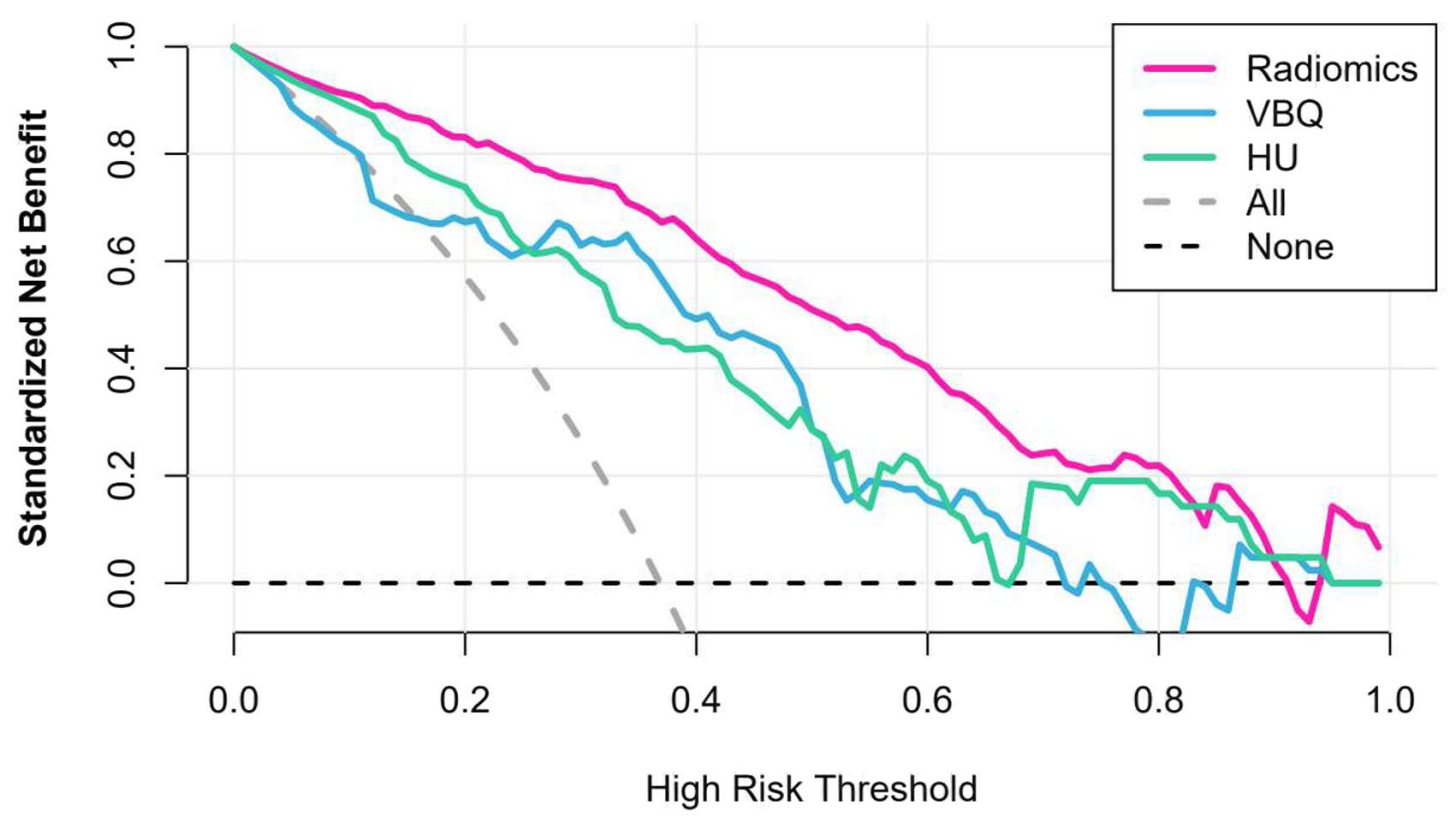
Decision curve analysis for each model. The *y*-axis displays the net benefit, a metric that balances clinical value by combining true-positive detections (benefits) and penalizing false-positive outcomes (harms). The harms are scaled by a factor reflecting the relative severity of missing a true osteoporotic lesion versus the risks of unnecessary treatment. The gray line assumes all lesions are osteoporotic (avoiding treatment entirely). The black line assumes no lesions are osteoporotic (treating all cases).

DCA for the radiomics model, VBQ score and HU value is presented in Figure 6. The radiomics model demonstrated significantly greater net clinical benefit than both VBQ and HU approaches when compared with no-model clinical strategies (treat-all or treat-none) across diagnostic probability thresholds.

## Discussion

With the growing aging population, the prevalence of osteoporosis is rapidly increasing, becoming a significant public health issue worldwide. OVCF is a typical manifestation of osteoporosis, which can cause chronic pain, spinal kyphosis and long-term disability. To assess preoperative bone quality, DEXA is considered the gold standard for diagnosing osteoporosis. Another emerging BMD measurement technology is quantitative CT scanning (QCT), but QCT is difficult to be extensively used due to the limitation of equipment and software. In addition, the clinical application of QCT is often hindered by high economic costs. Since QCT in our hospital is not routinely applied, we used DEXA as the standard for diagnosing osteoporosis in present study. However, recent studies have reported that degenerative diseases, including osteophytes, osteosclerosis, and severe disc herniation, can significantly affect the accuracy of the results (7, 21). In view of this situation, a diagnostic method with high accuracy is still needed to reflect BMD.

Radiomics is an emerging interdisciplinary field with broad application prospects and challenges, expected to advance the development of precision medicine. Some studies have shown that radiomic models based on lumbar spine CT images (14, 22) and MRI (T1WI + T2WI) (15) can identify patients with osteoporosis. Machine learning is an artificial intelligence technology that analyzes and filters complex, unordered data through algorithms to guide clinical decision-making. The combination of radiomics and machine learning technology is an emerging, non-invasive and reproducible method for artificial intelligence image-assisted diagnosis (23–25). Earlier study has proposed that machine learning algorithm models combining lumbar X-ray images with specific clinical risk factors demonstrate ideal predictive performance in diagnosing osteoporosis (26).

In this study, we identified that the radiomic signature derived from CT imaging features serves as a valuable biomarker for diagnosing osteoporosis. Compared to traditional visual analysis, radiomics can provide more accurate and objective bases for qualitative and quantitative disease analysis through mathematical algorithms (27). Seven classes of quantitative radiomics features based on the spine CT have been extracted, including the first-order features, shape features, GLCM features, GLDM features, GLRLM features, GLSZM features and NGTDM features. These sequences provide quantitative images of bone marrow in multiple dimensions, accurately reflecting the microstructure of the bone marrow. Furthermore, our comprehensive analysis showed that radiomics features selected based on repeatability and redundancy contribute to the most valuable features. Specifically, features with excellent inter- and intra-observer reliability (ICC >0.9) were selected for redundancy reduction by MRMR and LASSO methods. Eight hundred and fifty-one selectable radiomic features were condensed into nine features in the radiomic signature model development. Combined with imaging features in radiomics model, patients can be successfully divided into low-score group and high-score group, and there is a significant difference in the probability of osteoporosis. The predictive models established and verified by using machine learning algorithms including LR, SVM, XGBoost and RF exhibit good diagnostic performance. The radiomics-XGBoost model demonstrated superior predictive accuracy, achieving AUC values of 0.891 in the training cohort and 0.910 in the validation cohort. The calibration curve shows a high degree of fit between the predicted probabilities of the radiomics-XGBoost model and the actual probabilities, indicating good predictive performance. We believe that the proposed CT-based machine learning radiomics model can assist clinical decision-making by predicting osteoporosis, especially in cases where DEXA or QCT is unavailable.

The interpretability of the selected radiomic features lends crucial biological plausibility to our model. These features are not merely mathematical abstractions but likely reflect fundamental pathophysiological changes in osteoporotic bone. Specifically, the alteration in first-order skewness may indicate a shift in bone mineral distribution, while the textural changes captured by GLCM cluster shade and correlation suggest a disruption of the homogeneous trabecular microstructure. The significance of GLSZM features is particularly telling: an increase in LargeAreaLowGrayLevelEmphasis may correspond to expanded regions of bone resorption and marrow space, whereas SmallAreaEmphasis could reflect the fragmentation of trabecular plates. Collectively, this radiomic signature provides a composite, quantitative profile that encapsulates both the diminished bone density and the degraded microarchitectural quality characteristic of osteoporosis, thereby bridging the gap between high predictive performance and meaningful clinical interpretation. Critically, this degree of interpretability directly addresses a key barrier to clinical adoption of machine learning models. By mapping abstract features to established pathological concepts, the model’s predictions become more transparent and trustworthy to clinicians. When a model’s decision can be rationalized in terms of recognizable disease processes, clinicians are more likely to understand, trust, and ultimately integrate its output into their diagnostic reasoning or risk assessment workflow.

Currently, the measurement of CT HU value and MRI VBQ score are recommended as alternative methods to represent BMD. Hocaoglu et al. (28) demonstrated that HU value was positively correlated with lumbar bone density, and the established threshold may be a promising tool for diagnosing osteoporosis. Additionally, Yin et al. (29) found that the VBQ score is closely related to BMD and can be used to assist in the diagnosis of osteoporosis. A recent study indicated that combining HU values and VBQ scores in preoperative screening significantly improves accuracy compared to individual assessments (30). Therefore, it is reasonable to compare the radiomics-based machine learning model to the VBQ and HU method. Both the DeLong test and DCA analysis in our present study indicated that the radiomics model outperformed the VBQ and HU model in detecting osteoporosis, demonstrating a high ability to differentiate osteoporosis.

While the radiomics-based model can serve as an auxiliary tool for screening osteoporosis, researchers should acknowledge the existing gap between research findings and clinical application. External generalizability and the automation of segmentation are two significant challenges hindering the translation of radiomics into clinical practice (31). Although our study incorporated an internal test cohort, additional independent validation datasets are essential to confirm the scalability of our models for widespread clinical use. As automatic segmentation technology advances, feature extraction and computation can be seamlessly integrated into unified software solutions, potentially streamlining radiomics into a single-click operation in the future. It is important to note that the proposed radiomics model is envisioned as a screening, not a diagnostic, tool. A positive “osteoporotic” prediction should trigger confirmatory testing with DEXA and a comprehensive clinical assessment.

The current study has some limitations. First, a limitation of our current binary model is its inability to specifically stratify patients with low bone mass (osteopenia), a cohort whose management often depends on additional risk factors. Second, the data from this study is based on a small single-center sample, necessitating prospective multi-center studies to further validate our results. It is important to note that this study utilized a retrospective dataset from a single institution, with CT scans acquired on a single scanner using a fixed protocol. While this approach ensured homogeneity for our radiomic feature extraction and model development, it may limit the model’s performance when applied to external cohorts with different acquisition parameters, such as tube voltage, reconstruction kernel, and contrast timing. Third, deep learning has significant potential in the field of medical imaging, as it can automatically identify and classify features from target images, and it has been applied in osteoporosis research (32, 33); however, it was not explored in this study. Then, this study did not distinguish between the use of *T*-score and *Z*-score, which may have underestimated the bone density level of a small number of young patients. Therefore, the current findings are more applicable to middle-aged and elderly patients.

## Conclusion

The present results show the promising potential of machine learning radiomics analysis based on lumbar CT in detecting osteoporosis. XGBoost model had the best predictive performance and can better help clinicians to diagnose osteoporosis. In addition, the machine learning radiomics model outperformed the VBQ and HU model. In the future, further external testing data using multicenter and large samples are needed to confirm the current study.

## Data Availability

The raw data supporting the conclusions of this article will be made available by the authors, without undue reservation.

## Glossary

DEXA: Dual-energy X-ray absorptiometry
CT: Computed tomography
HU: Hounsfield unit
OVCF: Osteoporotic vertebral compression fractures
MRI: Magnetic resonance imaging
VBQ: Vertebral bone quality
ASD: Adjacent segment disease
ROI: Region of interest
BMI: Body mass index
BMD: Bone mineral density
GLCM: Gray level co-occurrence matrix
GLDM: Gray level dependence matrix
GLRLM: Gray level run length matrix
GLSZM: Gray level size zone matrix
NGTDM: Neighborhood gray tone difference matrix
ICC: Intraclass correlation coefficient
MRMR: Minimum redundancy-maximum relevance
LASSO: Least absolute shrinkage and selection operator
LR: Logistic regression
SVM: Support vector machines
XGBoost: Extreme gradient boosting
RF: Random forest
ROC: Receiver operating characteristic
AUC: Area under the curve
DCA: Decision curve analysis
